# Bio-removal of rare earth elements from hazardous industrial waste of CFL bulbs by the extremophile red alga *Galdieria sulphuraria*

**DOI:** 10.3389/fmicb.2023.1130848

**Published:** 2023-02-13

**Authors:** Anjali Singh, Mária Čížková, Vít Náhlík, Dana Mezricky, Dominik Schild, Marian Rucki, Milada Vítová

**Affiliations:** ^1^Laboratory of Cell Cycles of Algae, Centre Algatech, Institute of Microbiology, Czech Academy of Sciences, Třeboň, Czechia; ^2^Faculty of Fisheries and Protection of Waters, South Bohemian Research Center of Aquaculture and Biodiversity of Hydrocenoses, Institute of Aquaculture and Protection of Waters, University of South Bohemia, České Budějovice, Czechia; ^3^Institute of Medical and Pharmaceutical Biotechnology, IMC FH Krems, Krems, Austria; ^4^Laboratory of Predictive Toxicology, National Institute of Public Health, Prague, Czechia; ^5^Centre for Phycology, Institute of Botany, Czech Academy of Sciences, Třeboň, Czechia

**Keywords:** compact fluorescent lamp, industrial wastes, extremophile, Rhodophyta, *Galdieria sulphuraria*, bio-removal, plant hormones

## Abstract

In recent decades, a shift has been seen in the use of light-emitting diodes over incandescent lights and compact fluorescent lamps (CFL), which eventually led to an increase in wastes of electrical equipment (WEE), especially fluorescent lamps (FLs) and CFL light bulbs. These widely used CFL lights, and their wastes are good sources of rare earth elements (REEs), which are desirable in almost every modern technology. Increased demand for REEs and their irregular supply have exerted pressure on us to seek alternative sources that may fulfill this demand in an eco-friendly manner. Bio-removal of wastes containing REEs, and their recycling may be a solution to this problem and could balance environmental and economic benefits. To address this problem, the current study focuses on the use of the extremophilic red alga, *Galdieria sulphuraria*, for bioaccumulation/removal of REEs from hazardous industrial wastes of CFL bulbs and the physiological response of a synchronized culture of *G. sulphuraria*. A CFL acid extract significantly affected growth, photosynthetic pigments, quantum yield, and cell cycle progression of this alga. A synchronous culture was able to efficiently accumulate REEs from a CFL acid extract and efficiency was increased by including two phytohormones, i.e., 6-Benzylaminopurine (BAP - Cytokinin family) and 1-Naphthaleneacetic acid (NAA - Auxin family).

**GRAPHICAL ABSTRACT:**
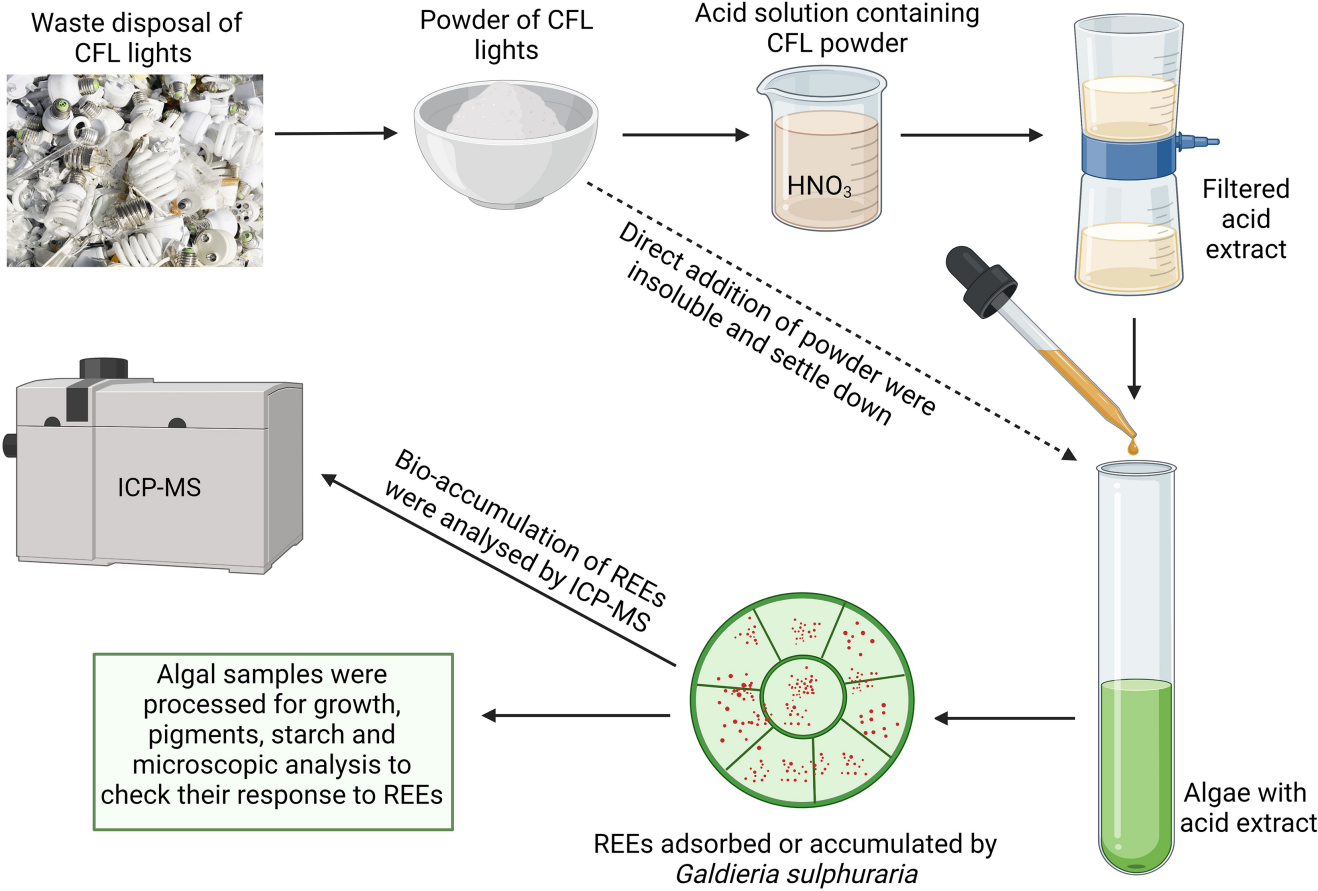


## Introduction

Rare earth elements (REEs) are a group of 17 chemical elements that comprise yttrium (Y), scandium (Sc), and a series of 15 lanthanides. REEs have practically identical physical and chemical properties although they have rather unique magnetic and catalytic properties (Cheisson and Schelter, 2019; Čížková et al., 2021). These properties make them desirable in a wide range of industries such as electrical, electronics, laser, glass, magnetic materials, energy technology, aquaculture, and agriculture (Hu et al., 2004; Chakhmouradian and Wall, 2012; Balaram, 2019). In recent decades, the use of fluorescent lamps (FLs), involving both tubes and compact fluorescent lamps (CFLs), has increased globally to improve energy efficiency. However, due to the presence of mercury and REEs as essential components, their use can be environmentally harmful (Balaram, 2019; Pagano et al., 2019). The shift from incandescent lights and CFLs to light-emitting diodes has generated a considerable amount of waste electrical equipment (WEE) (Baldé et al., 2017; Gwenzi et al., 2018; Pagano et al., 2019). Among the most common elements present in WEE are the REEs yttrium (Y), europium (Eu), and terbium (Tb). These economically important REEs are usually discarded into our environment as wastes, having a consequential effect on the linear flow of goods throughout the economy (Balaram, 2019). Increased demands for REEs, has exerted pressure on industrialized countries to look for alternatives to fulfill their demand in an eco-friendly manner. Bio-removal of WEE containing REEs, and their recycling can have a positive impact, balancing both environmental and economic benefits.

The role of plants, microbes (i.e., bacteria, cyanobacteria, and fungi), and algae as REE accumulators, bio-removers, and potential bio-miners have been studied extensively (Dubey and Dubey, 2011; Qu and Lian, 2013; Pinto et al., 2020; Jalali and Lebeau, 2021). Among these, algae-based bioaccumulation/bio-removal is considered to be one of the most promising methods due to its high efficiency, wide applicability and the low-cost recovery. However, mostly contaminated water, soil or red mud were used as secondary sources of REEs (Pinto et al., 2020; Lima and Ottosen, 2021). Regarding WEE (e.g., CFL lights) as secondary source of REEs, only a few studies have demonstrated the use of microalgae as potential REE accumulators/sorbents or removers (Čížková et al., 2021). However, several studies have been conducted that demonstrate the growth of green and red algae in the presence of a single REE, or waste containing a mixture of REEs (Čížková et al., 2021; Pinto et al., 2021). The growth of green algae *Chlamydomonas reinhardtii*, *Chlorella* sp. or *Arthrospira* sp. improved in the presence of REEs (Liu and Shizong, 1999) whereas, another study showed that due to the presence of multiple REEs in CFL powder, *Galdieria* growth and dry matter accumulation slowed compared to the control (Čížková et al., 2021).

In addition, extensive research has been conducted to investigate heavy metal (HM) bioaccumulation/bioremediation by algae employing different chemical agents and phytohormones. However, the underlying mechanisms controlling the effect of phytohormones on bioremediation are elusive (Piotrowska-Niczyporuk et al., 2012, 2018). Unlike HM bioremediation, fewer studies have been conducted to understand REE bio-removal/bioaccumulation (Pinto et al., 2020; Čížková et al., 2021; Pinto et al., 2021). Literature describing the use of chemical agents commonly employed in bioremediation, or phytohormones that affect the biosorption/bio-removal of REEs are also scarce. This study is the first showing the use of two phytohormones, 6-Benzylaminopurine (BAP - Cytokinin family, known to affect cell division) and 1-Naphthaleneacetic acid (NAA - Auxin family, generally known as growth stimulators) on REE accumulation by the red algae *G. sulphuraria*.

Recently, algae from the extremophile group of cyanidiophyceae, especially the unicellular red alga *Galdieria*, has been proposed as suitable models that have the potential to accumulate or adsorb different REEs (Minoda et al., 2015; Čížková et al., 2019, 2021). *G. sulphuraria* grows under thermo-acidophilic conditions, and inhabits hot sulfur springs, toxic metal-containing and geothermal habitats (Gross et al., 1998; Yoshimura et al., 1999; Reeb and Bhattacharya, 2010; Minoda et al., 2015). The organism is known to thrive over in moderate/high temperatures ranging from 35°C to 56°C (Reeb and Bhattacharya, 2010; Carfagna et al., 2015; Bottone et al., 2019), and pH ranging from 0.2 to 6 (Yoon et al., 2006; Náhlík et al., 2021; Abiusi et al., 2022). Its resistance to toxic metals and REEs is exceptional among other eukaryotic algae, thus it is a suitable organism to achieve bioaccumulation/bio-removal of metals from waste material (Yoshimura et al., 1999; Minoda et al., 2015; Čížková et al., 2021). Unlike other microalgae, *G. sulphuraria* produces highly branched and low molecular weight glycogen as an energy and carbon reserve instead of starch supplying energy for processes related to cell multiplication such as DNA replication, nuclear division, cytokinesis, daughter cell formation and release of autospores (Vítová et al., 2015; Martinez-Garcia et al., 2017). The accumulation of starch during the cell cycle, and its consumption during the cell division in dark phase has been described in several synchronized cultures of green algae (Brányiková et al., 2011; Vítová et al., 2015). Recently, Náhlík et al. studied the accumulation of glycogen in *G. sulphuraria* throughout the cell cycle following light and dark phases. Results showed that some accumulated glycogen was consumed during cell division (Náhlík et al., 2021). Like other microalgae, *G. sulphuraria* divides by multiple fission and releases 2^n^ autospores from a single mother cell within one cell cycle (Jong et al., 2021).

One of the important fluorescence parameters followed in photosynthetic microalgae is the F_v_/F_m_ ratio, a valuable bio-indicator for overall photosynthetic performance, indicating maximal photochemical quantum efficiency of photosystem II (PSII). The F_v_/F_m_ index also indicates stress conditions such as temperature, light, pH, nutrient concentration, or toxic metals (Schreiber et al., 1986; Iovinella et al., 2020). The ratio of F_v_/F_m_ differs in photosynthetic organisms from plant to cyanobacteria, in land plants F_v_/F_m_ varies between ∼0.75–0.8, and slightly lower values ∼0.7 were reported in green algae. However, red algae and cyanobacteria have significantly lower values of F_v_/F_m_ ratio between ∼0.5–0.6 and ∼0.2–0.4, respectively (Oesterhelt et al., 2007; Iovinella et al., 2020). Like photosynthetic efficiency, photosynthetic pigments are also vulnerable to stress conditions. Until now, the effect of REEs on photosynthetic pigments have not been investigated thoroughly, although research has shown that exposure to Lu^3+^ (lutetium) and Sc^3+^ (scandium) significantly reduced levels of photosynthetic pigments in *Parachlorella kessleri* (Goecke et al., 2017).

In the present study, we describe the effect of a CFL acid extract on a synchronous culture of *G. sulphuraria* throughout the cell cycle. Furthermore, accumulated glycogen and the photosynthetic pigment profile of a synchronously growing and dividing culture were analyzed. This study provides insight into the impact of REEs on the cell cycle of *G. sulphuraria*. The study also determined how efficiently this red alga could accumulate REEs from a CFL extract and the effect of two synthetic plant hormones (BAP and NAA).

## Materials and methods

### Algal culture and growth conditions

The experimental organism, unicellular red alga *G. sulphuraria* (Galdieri) Merola, 002 was acquired from the Algal Collection of the University “Federico II” of Naples, Italy.^1^ In general, algal cells were cultivated photoautotrophically in a Galdieria-nutrient medium (modified Allen medium) prepared to the following final composition of macroelements (g L^−1^): 1.31 (NH_4_)_2_SO_4_, 0.27 KH_2_PO_4_, 0.25 MgSO_4_·7H_2_O, 0.02 C_10_H_12_O_8_N_2_NaFe, 0.14 CaCl_2_·2H_2_O, and microelements diluted 500x from the stock solution (mg L^−1^): 31 H_3_BO_3_, 1.25 CuSO_4_·5H_2_O, 22.3 MnSO_4_·4H_2_O, 0.88 (NH_4_)_6_Mo_7_O_24_·4H_2_O, 2.87 ZnSO_4_·7H_2_O, 1.46 Co(NO_3_)_2_·6H_2_O, 0.014 V_2_O_4_(SO_4_)_3_·16H_2_O, 0.3 Na_2_NO_4_·7H_2_O, 1.19 KBr, 0.83 KI, 0.91 CdCl_2_, 0.78 NiSO_4_, 0.12 CrO_3_, 4.74 Al_2_(SO_4_)_3_K_2_SO_4_·24H_2_O (all chemicals from Penta, Chrudim, Czech Republic) in distilled water, autoclaved for 20 min. The synchronization of the cultures was carried out by changing the light and dark periods (16 l/8D) according to Náhlík et al. (2021). The culture was cultivated under optimal conditions of pH 3, temperature of 40°C, and light intensity of 350 μmol photons m^−2^ s^−1^. The photobioreactors, in the shape of glass cylinders (300 ml) or flat cuvettes (2.5 l), were placed in a thermostatic water bath and illuminated by a panel of dimmable fluorescent lamps (OSRAM DULUX L55 W/950 Daylight, Milano, Italy). Algal culture suspensions were supplied with a gas mixture of air and CO_2_ (2% v/v), at a flow rate of 15 L h^−1^. The experiments were carried out in a batch culture regime under controlled light and temperature conditions.

### Preparation of CFL acid extract

Luminophore powder from e-waste of CFL light bulbs was provided by RECYKLACE EKOVUK a.s. (Příbram, Czech Republic). The particle size of CFL powder was 25 μm^3^ and it was insoluble in water or Galdieria-nutrient medium. Based on the preliminary study with different acids, acid mixtures, and their concentrations, the best solubility of CFL particles was observed in 10% HNO_3_ acid. To prepare the CFL acid extract with a final concentration of 40 μg mL^−1^, 4 g of CFL powder was extracted into 100 ml of 10% HNO_3_. The solution was shaken on a horizontal shaker (at 150 rpm) for 1 h and then extracted at room temperature for 12 h, followed by 3 h of shaking. The suspension was centrifuged at 4000 rpm for 3 min and the supernatant was filtered through a 0.45 μm filter to avoid the remaining solid particles. The resulting clear CFL acid extract solution has an approximate pH of 0.5. The stock solution was used in all experiments.

### CFL acid extract treatment

To study the effect of CFL extract on various parameters of the cell cycle and the accumulation of REEs, 4% CFL acidic extract (v/v) was used to treat the algal culture. After adding the CFL extract to nutrient medium, the pH dropped to 2, and was adjusted with approximately 0.5 ml of NH_4_OH to pH 3. For CFL treatment, the synchronous cultures of *G. sulphuraria*, at an initial concentration of 1×10^6^ cells L^−1^, were cultivated in 4% of CFL extract for 24 h. Samples of the algal culture were collected at desired time intervals for further analyses. Along with CFL–treated culture, a control (untreated culture) was also inoculated with the same initial culture concentration and incubated for 24 h.

### Plant hormone treatment

To study the effect of hormones on REEs accumulation, one set of asynchronous cultures of *G. sulphuraria* was cultivated with CFL and hormones for 24 h. For this study two synthetic plant hormones, 6-Benzylaminopurine (BAP-Cytokinin family) and 1-Naphthaleneacetic acid (NAA-Auxin family) (Sigma-Aldrich) were used at a final concentration of 5 mg L^−1^ for this study. At the end of the experiment, cultures with and without plant hormones were harvested by centrifugation (3,000 rpm, 5 min), freeze dried and analyzed by ICP-MS.

### Dry matter and doubling time determination

Dry matter was determined from 5 ml of algal suspension centrifuged at 4000 rpm for 5 min in dried and pre-weighed 5 ml test tubes. The pellet was dried at 105°C for 24 h and weighed on a Sartorius TE214S-0 CE analytical balance.

Doubling time (DT) was calculated for both the control and treated cultures of *G. sulphuraria* based on dry matter (DM) according to the equation $DT\left(h\right)=\frac{\left(T_{t}-T_{0}\right)*\log\left(2\right)}{\left(\log\left(DM_{t}\right)-\log\left(DM_{0}\right)\right)}$ , where T_t_ is the time of the end of the light phase, T_0_ is the starting time, and DM_t_ is the value of dry matter at T_t_ and DM_0_ is the value of dry matter at T_0_.

### Pigment analysis

To determine the content of chlorophyll *a* (Chl *a*) and carotenoids (car), 10 ml of homogenized suspension of both the control and CFL–treated cultures were centrifuged at 4000 rpm for 5 min. Harvested pellets were suspended in 1 ml of phosphate buffer (containing 0.1 M KH_2_PO_4_: 0.1 M Na_2_HPO_4_·12H_2_O, 1:9; pH 7.7) and 10 μg of MgCO_3_.The pellets were then each mixed with 500 μl of 0.75–1.00 mm glass beads (P-LAB, Prague, Czech Republic) and vortexed for 5 min to break the cell walls. For pigment extraction, 4 ml of 100% acetone was added, mixed well, and centrifuged at 4000 rpm for 5 min. After the first round of extraction, the supernatant was transferred to a calibrated tube with a stopper and placed in a dark block. The extraction process was repeated with 80% acetone and the supernatant was transferred to the same calibrated tube. The final volume of extract was made up to 10 ml using 80% acetone. The absorbance of the solution was recorded at 750, 664, 647, 470, and 450 nm by UV-1800 spectrophotometer, Shimadzu Corporation (Kyoto, Japan). The content of chlorophyll *a* and carotenoids were calculated according to Wellburn (1994).

Phycobiliproteins were extracted in 10 ml of homogenized control and CFL–treated culture samples. Each culture was centrifuged at 4000 rpm for 10 min at room temperature and the pellet was washed twice with distilled water. Finally, the culture was re-suspended in 20 mM acetate buffer (pH 5.1) containing 40 mM NaCl and 0.02 M sodium azide, followed by bead beating and repeated freeze-thawing until the phycobiliproteins were released into the supernatant. The collected supernatant was measured in a UV–VIS spectrophotometer (Shimadzu 1800-UV, Shimadzu Corp., Japan). The estimation of phycobiliproteins, expressed in mg mL^−1^,was carried out by following the equations of Bennett and Bogorad (1973) and Johnson et al. (2014). All chemicals were supplied by Penta (Chrudim, Czech Republic).

### Photosynthesis evaluation

Photosynthetic activity was evaluated by fluorimeter as the quantum yield (F_v_/F_m_). Two milliliters aliquots were withdrawn from the culture and placed into 10 × 10-mm plastic cuvettes for 30 min in the dark. Quantum yield was measured using an AquaPen-C 100 PAM fluorimeter (Photon Systems Instruments, Drasov, Czech Republic).

### Determination of glycogen content

Glycogen content was determined by the anthrone method (McCready et al., 1950) according to the modified protocol of Brányiková et al. (2011). Briefly, 10 ml algal samples were harvested by centrifugation at 3000 rpm for 3 min and the cell pellet was stored at −20°C. 250 μl zirconium beads (0.7 mm diameter) and 500 μl dH_2_O were added to thawed samples and vortexed (Vortex Genie 2, Scientific Industries, Inc., Bohemia, NY, United States) for 5 min at 3,200 rpm for cell breakage. For depigmentation of algae, 1 ml of 80% ethanol was added to the sample then vortexed and incubated at 68°C for 15 min in a water bath. This process was repeated 3–4 times until the pellet was clear (green color free). The glycogen-containing cell pellets were hydrolyzed with 1.5 ml of 30% perchloric acid for 15 min at room temperature, then centrifuged and supernatants were collected into pre-prepared calibration test tubes. This process was repeated twice more, and the extracts were combined and made up to 5 ml using 30% HClO_4_. The colorimetric reaction was then carried out by mixing 500 μl of ice-cold extract with 2.5 ml of anthrone reagent (2 g of anthrone in 1 l of 72% (v/v) ice-cold sulphuric acid). The mixture was boiled at 100°C for 8 min followed by cooling and quantification at 625 nm (A_625_) using a Shimadzu UV-spectrophotometer UV-1800 (UV-1800, Shimadzu, Kyoto, Japan). The same procedure was followed for the blank (500 μl of 30% HClO_4_) and standard tubes (500 μl of glucose (100 mg L^−1^) in 30% HClO_4_). Glycogen calibration was carried out simultaneously using glucose as the standard. To obtain the calibration curve for glycogen determination, the values measured for glucose were multiplied by 0.9. The data were expressed as picograms (pg) of glycogen per cell.

### Confocal microscopy

Confocal images of treated and control cells were captured with an inverted Zeiss LSM 880 laser scanning confocal microscope (Carl Zeiss Microscopy GmbH, Oberkochen, Germany) equipped with a Plan-Apochromat 63x/1.4 NA Oil DIC M27 immersion objective. SYBR-Green was excited by Argon laser 488 nm (laser power 0.03%), its emission was captured by GaAs-detector at wavelengths 499–571 nm, Gain 750. Likewise, chlorophyll auto-fluorescence was excited by Argon laser 488 nm (laser power 0.15%) and detected at 695–759 nm (PMT detector, using photon counting mode). This track was on top of that used to create T-PMT signal (transmission) using Gain 370. The pinhole for this excitation wavelength was kept at 69 μm diameter. MBS was chosen 488 and pixel dwell time was given 16.38 μs. The images were then processed using ImageJ software.

### Statistical analysis

All experiments were performed in three biological replicates (*n* = 3). The presented results are the averages and standard deviations from all three replicates. The data statistics analysis was generated using the Real Statistics Resource Pack software (Release 8.4) for MS Excel 2013. Copyright (2013–2021) Charles Zaiontz^2^ (accessed on 3 January 2023). To quantify the relationship between predictor variables (time, treatment) and a response variable (concentration of pigments, or concentration of glycogen, or F_v_/F_m_ ratio) multiple linear regression model was used. The fitted regression model was: ß1 + (ß2*time) + (ß3*treatment) + (ß4*time^2) + (ß5*(time*treatment)), where the coefficient values correspond to predictors: ß1 - Intercept, ß2 - Time, ß3 - Treatment, ß4-Time^2, ß5 – Time*Treatment. The comparison of individual REE concentration levels at different time points or between experimental treatments (CFL, CFL + hormones) was performed using the one-way ANOVA test and Tukey’s HSD test. A value of *p* < 0.05 was considered significant.

### Quantitative REE analysis by ICP-MS

Samples of CFL alone and CFL-treated algal biomass were digested with 30% H_2_O_2_ and 67% HNO_3_ (Merck, Suprapure) in a PTFE microwave oven (MLS1200 MEGA, Gemini bv, Apeldoorn, The Netherlands) at 250–600 W for 20 min. Quantitative analysis of REEs was performed using an Elan DRC-e (Perkin Elmer, Concord, ON, Canada) which is equipped with a concentric PTFE nebulizer and cyclonic spray chamber. Algal samples were passed through a 0.45 μm nylon syringe filter (Millipore, Molsheim, France) and diluted 1:10 with distilled water. Values were expressed as micrograms per gram dry weight (μg g^−1^ DM).

## Results

Before conducting the experiments and because CFL was extracted into 10% HNO_3_, the effect of 10% HNO_3_ on a synchronous culture of *G. sulphuraria* was studied. Results of this preliminary study showed that there was no negative effect of 10% HNO_3_ on the cell shape, size, or growth of this organism (Supplementary Figures S1, S2).

### Effect of CFL on growth of *Galdieria sulphuraria*

**Figure 1:**
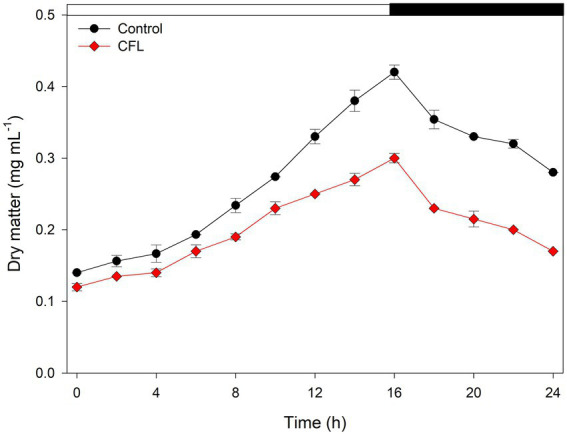
Growth of synchronous culture of *Galdieria sulphuraria* in control (black circles) and CFL–treated (red diamonds) medium, at pH 3, a light intensity of 350 μmol photons m^−2^ s^−1^ and a temperature of 40°C, expressed as dry matter (DM) in μg mL^−1^. The dark phase is indicated by the black bar above the graph. The data are plotted as means of biological triplicates. The error bars represent standard deviations (±SD) and are shown when larger than the symbol size.

Growth of the control culture of *G. sulphuraria* and the culture treated with CFL extract was expressed as dry matter in mg mL^−1^ (Figure 1). Our results showed a progressive increase in dry matter of both control and treated cultures up to 16 h (light regime), after which, a decline was observed due to a light limitation. The control culture of *G. sulphuraria* reached a dry matter content of 0.42 mg mL^−1^ during the light phase whereas the culture treated with CFL extract achieved only 0.30 mg mL^−1^ dry matter. The loss of dry matter from 0.42 mg mL^−1^ to 0.28 mg mL^−1^ in the control culture and from 0.30 mg mL^−1^ to 0.17 mg mL^−1^ in CFL–treated culture during the dark phase (Figure 1, dark phase) could be due to losses by respiration (night biomass loss). The calculated mass doubling time (see Methods for the equation) was 10.09 h ± 0.30 for the control culture and 12.10 h ± 0.48 for the culture treated with CFL extract.

### The effect of CFL on photosynthetic pigments

**Figure 2:**
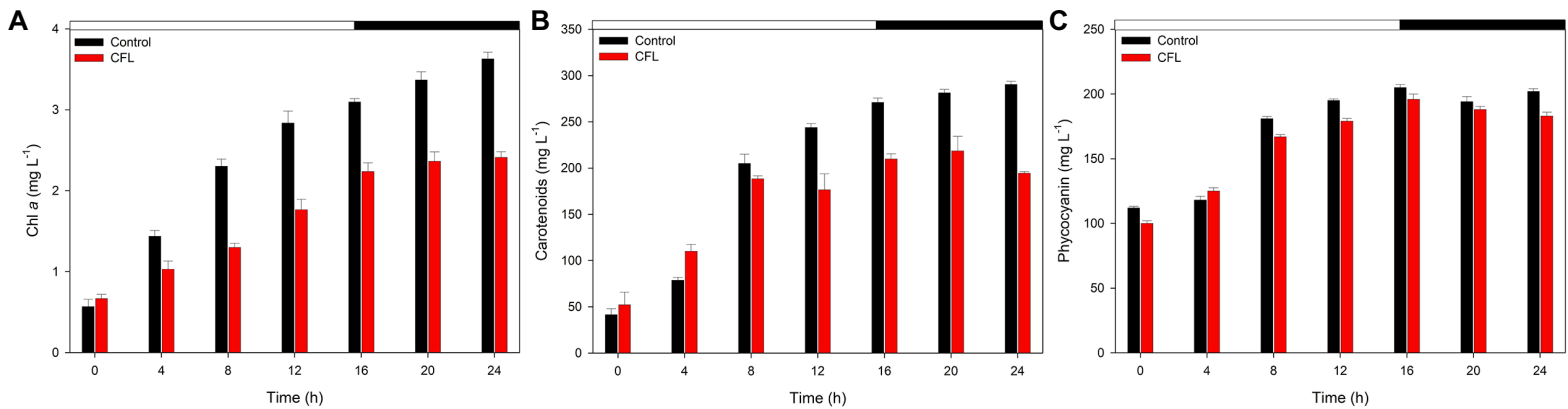
Photosynthetic pigments in synchronous control (black bar) and CFL–treated (red bar) *Galdieria sulphuraria* cultures, expressed as mg L^−1^ **(A)** chlorophyll *a*, **(B)** carotenoids, **(C)** phycocyanin. The dark phase is indicated by the black bar above the graph. The data are plotted as means of biological triplicates. The error bars represent standard deviations (±SD). For details of statistical analysis (multiple linear regression) see Supplementary Table S1.

The effect of CFL extract on chlorophyll *a*, carotenoids, and phycocyanin contents was observed at 4 hourly intervals over the period from 0 to 24 h synchronous culture. Figure 2 shows the comparative effect of CFL–treated and control cultures on pigment content. Results showed that all three photosynthetic pigments progressively increased with increasing duration of the experiment, although CFL–treated cells showed lower pigment levels than the control culture. Figure 2A shows the synthesis of chlorophyll *a*, which was affected by CFL treatment throughout the experiment. At the end of the light phase (16 h), the Chl *a* content was recorded as 3.0 mg L^−1^ in the control culture whereas, in the CFL–treated culture, Chl *a* content was only 2.2 mg L^−1^. This loss in chlorophyll content could be due to CFL extract stress on the algae. In contrast, carotenoids, which are known to play a role in defense of algae against stress, increased till 4 h to 110 mg L^−1^ under CFL treatment compared with 78 mg L^−1^ in the control culture (Figure 2B). Later, the trend of values reversed, and at 16 h (end of the light phase), the CFL–treated culture yielded 209 mg L^−1^ carotenoids as compared to 270 mg L^−1^ in the control culture (Figure 2B). Like chlorophyll *a*, phycocyanin content also increased progressively throughout the experiment. The control culture showed a slightly higher level of phycocyanin at 16 h, i.e., 191 mg L^−1^ as compared to 176 mg L^−1^ in the CFL–treated culture (Figure 2C). Interestingly as compared to chlorophyll and carotenoids, phycocyanin was the pigment least affected by CFL acid extract stress.

Multiple linear regression was used to test if time (hours of cultivation) and treatment (control vs. CFL) significantly predicted the concentration of pigments (chlorophyll *a*, carotenoids, and phycocyanin) in the algal biomass (Figure 2). For fitted regression model see Methods. The overall regression was statistically significant for Chl *a* (*R*^2^ = 0.97, *F*(4, 9) = 132.56, *p* = 5.43E-08), carotenoids (*R*^2^ = 0.94, F(4, 9) = 61.55, *p* = 1.55E-06), and also for phycocyanin (*R*^2^ = 0.97, F(4, 9) = 125.25, *p* = 6.98E-08). Both time and time^2 significantly predicted the concentration of all three pigments. Treatment alone did not significantly predict their concentration, however, the interaction term time*treatment did significantly predict the concentration of chlorophyll *a*, and carotenoids. The treatment influence was therefore significantly time-dependent. There was no time-dependency for phycocyanin, and its concentration in both cultures was not significantly different. For β coefficients and corresponding *p*-values see Supplementary Table S1.

### The effect of CFL on photosynthesis efficiency (F_v_/F_m_)

**Figure 3:**
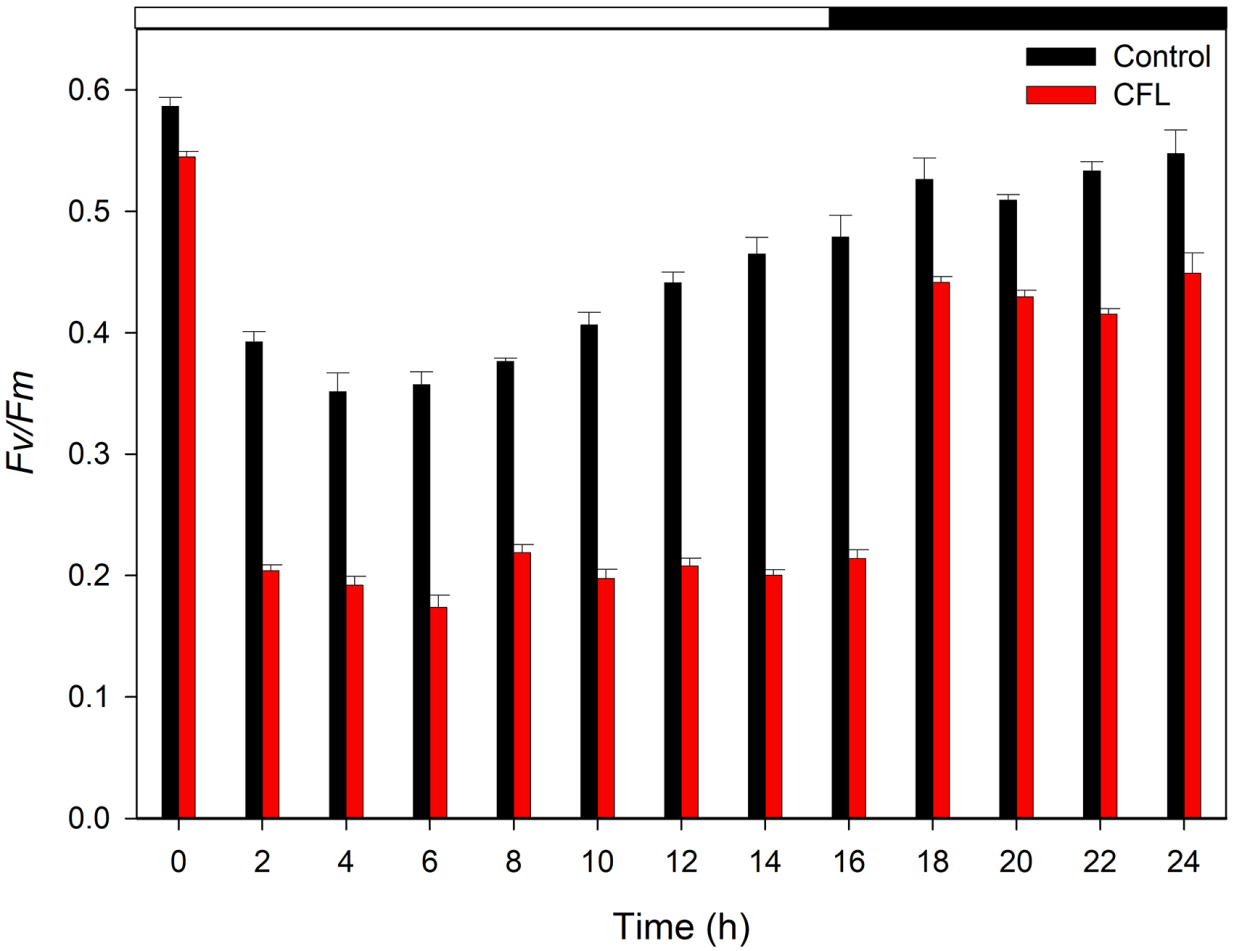
F_v_/F_m_ ratio of synchronous control (black bar) and CFL–treated (red bar) *Galdieria sulphuraria* cultures, showing the photochemical maximum quantum efficiency of PSII. The dark phase is indicated by the black bar above the graph. The data are plotted as means of biological triplicates. The error bars represent standard deviations (±SD). For details of statistical analysis (multiple linear regression) see Supplementary Table S1.

In the present study photosynthetic performance was determined as quantum yield (ratio F_v_/F_m_) which is the parameter commonly reflecting reduced function or impairment of the PSII reaction centers. At the beginning of the experiment, the F_v_/F_m_ ratio was recorded as 0.58 in the control culture and 0.55 in the CFL–treated culture (Figure 3). With progression of the cell cycle, at 6 h, the F_v_/F_m_ ratio fell to its minimum, i.e., 0.36 and 0.17 in the control and CFL–treated cultures, respectively, (light phase of Figure 3). This decrease in F_v_/F_m_ in the control culture recovered to almost its initial value, i.e., 0.53–0.56 at 18–24 h during the dark phase. The F_v_/F_m_ ratio in the CFL–treated culture recovered to a value of approximately 0.45, which is slightly lower than its original value (Figure 3, dark phase).

Multiple linear regression was used to test if time (hours of cultivation) and treatment (control vs. CFL) significantly predicted the F_v_/F_m_ ratio in the algal biomass (Figure 3). For fitted regression model see Methods. The overall regression was statistically significant (R^2^ = 0.65, *F*(4, 21) = 12.92, *p* = 1.84E-05). Both time and time^2 significantly predicted the F_v_/F_m_ ratio. Treatment alone significantly predicted the F_v_/F_m_ ratio, however, the interaction term time*treatment did not. For β coefficients and corresponding *p*-values see Supplementary Table S1.

### The effect of CFL on glycogen content

**Figure 4:**
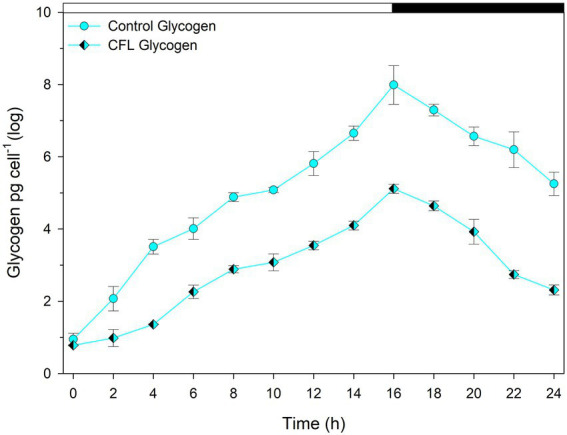
Concentration of total glycogen in synchronous *Galdieria sulphuraria* cultures. Control culture (cyan diamonds), CFL–treated culture (cyan-black diamonds). The dark phase is indicated by the black bar above the graph. The data are plotted as means of biological triplicates. The error bars represent standard deviations (±SD) and are shown when larger than the symbol size. For details of statistical analysis (multiple linear regression) see Supplementary Table S1.

The accumulation of glycogen in *G. sulphuraria* was followed throughout the cell cycle and samples were withdrawn every 2 h from both control and CFL–treated cultures (Figure 4). The net content of glycogen in both cultures progressively increased from the beginning of the cell cycle, showing the highest content, i.e., 8.0 pg. cell^−1^ in the control and 5.11 pg. cell^−1^ in the CFL–treated culture, at 16 h (at the end of the light phase). This represents and approximately 8-fold increase in glycogen in the control culture and 5.5-fold increase in the CFL-treated cells during the light phase; around 1/3 of the glycogen content was consumed during the dark phase in both cases (Figure 4).

Multiple linear regression was used to test if time (hours of cultivation) and treatment (control vs. CFL) significantly predicted the concentration of glycogen in the algal biomass (Figure 4). For fitted regression model see Methods. The overall regression was statistically significant [*R*^2^ = 0.92, *F*(4, 21) = 75.24, *p* = 3.84E-12]. Regarding the β coefficients and corresponding p-values in the Supplementary Table S1, it can be concluded that all predictor variables significantly predicted the concentration of glycogen. The treatment influence was significantly time-dependent, and the difference in glycogen concentration between control and CFL-treated culture was statistically significant.

### Course of the cell cycle

**Figure 5:**
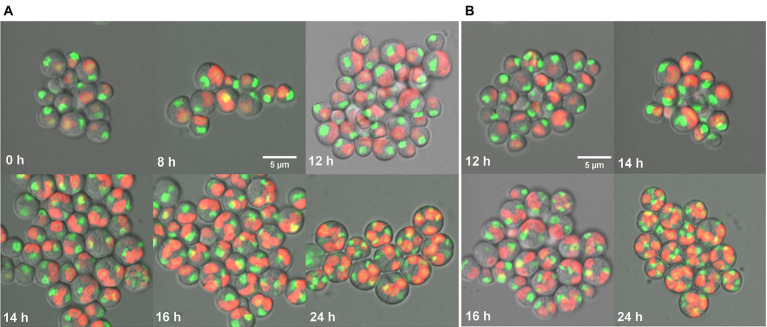
Fluorescent photomicrographs of cells of *Galdieria sulphuraria* under control conditions **(A)**, and in a CFL–treated culture **(B)**. Control culture: daughter cells - 0 h, growing single cells - 8 h, 1^st^ division of chloroplast and nuclei - 12 h, division into 2 cells - 14 h, division into 4 cells, protoplast division apparent - 16 h, four daughter cells growing inside the original mother cell wall before their release - 24 h. CFL–treated culture: chloroplast and nuclei started to divide - 12 h, apparent division into 2 cells - 14 h, started division into 4 cells and protoplast fission apparent - 16 h, four daughter cells growing inside the original mother cell wall before their release - 24 h. Nuclei in green were stained by SYBR Green I, chloroplasts in red - autofluorescence of chlorophyll. The bar represents 5 μm.

In this study, cell cycle development of *G. sulphuraria* was assessed by confocal microscopy using SYBR Green dye for nuclear staining (Figure 5). At the beginning of the cell cycle, during the light phase of the experiment, the single-nuclei daughter cells of both control (Figure 5A) and CFL–treated cultures (Figure 5B) were released from their mother cell walls (Figure 5A – 0 h). The cells started to grow in both cultures (Figure 5A – 8 h). In the control culture, chloroplasts and nuclei started to divide into two at 12 h (Figure 5A – 12 h). Protoplast division into two occurred at 14 h (Figure 5A – 14 h). At 16 h of the cell cycle, cells started to divide into four (Figure 5A – 16 h) which included the second chloroplast, nuclei, and protoplast fissions. At 24 h of the cell cycle, all the cells finished the division into four daughter cells, which remained by the mother cell wall (Figure 5A – 24 h). CFL-treated cells (Figure 5B) followed the same time course of the cell cycle as control cells, with the exception that the first cell division was delayed by 1–2 h (Figure 5B 12 h, 16 h).

### The effect of CFL on accumulation of REEs in algal biomass

**Figure 6:**
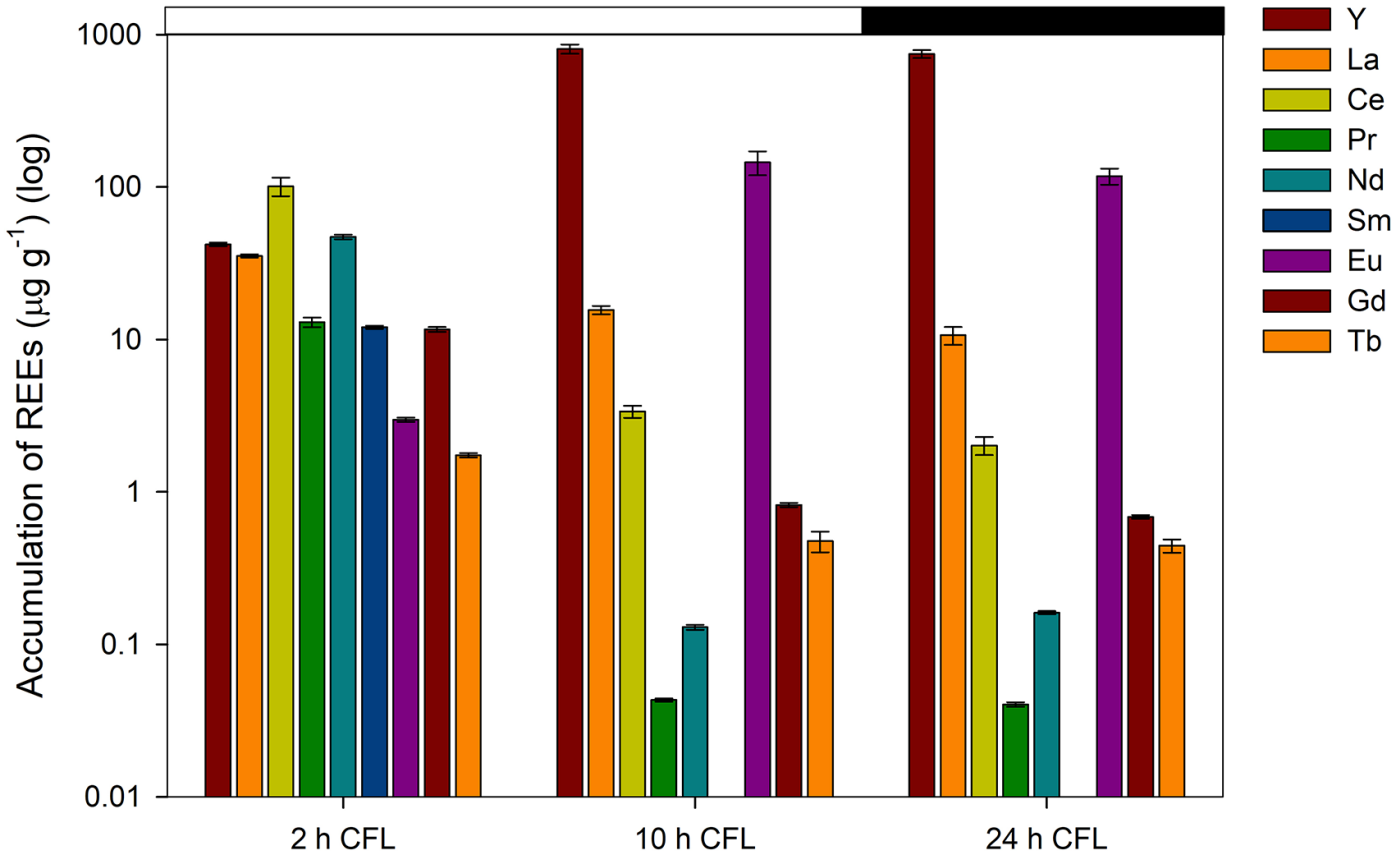
Accumulation of individual REEs in synchronous cultures of *Galdieria sulphuraria* under CFL–treatment expressed as concentration in μg g^−1^. The Y axis has a logarithmic scale. The X axis represents three different phases of the cell cycle (growth phase, commitment point, end of the cell cycle). The dark phase is indicated by the black bar above the graph. The error bars represent standard deviations (±SD). For details of statistical analysis (one-way ANOVA, Tukey’s HSD) see Supplementary Table S2.

ICP-MS analysis was conducted to observe the accumulation of REEs by a synchronous culture of *G. sulphuraria*. REE levels in the CFL acid extract were also analyzed using ICP-MS. The most abundant elements in the extract were La, Y, and Ce followed by Tb, Eu and Gd, respectively (Table 1). The results showed that specific REEs accumulated differently at different phases of the cell cycle. For example, at 2 h of the cell cycle (growth phase), Y and Eu levels were recorded as 42 and 2.7 μg g^−1^ DM respectively, which increased to 803 μg g^−1^ and 145 μg g^−1^ DM, respectively, at 10 h of the cell cycle (commitment point before the first division) (Figure 6). In contrast, at 2 h of the cell cycle, cerium (Ce) and La levels were 101 and 35 μg g^−1^ DM respectively, which drastically decreased to 2 and 10 μg g^−1^, respectively, at 24 h of the cell cycle (end of the cell cycle; cell division finished) (Figure 6). The most abundant lanthanides accumulated by *G. sulphuraria* were Y followed by Eu, La, and Ce (Figure 6). However, the uptake of REEs was not related to their abundance in the CFL acid extract (compare Figure 6; Table 1). There is an apparent increase in the content of Y and Eu in the biomass during the cell cycle, while a decrease of La and Ce was detected. Although Tb and gadolinium (Gd) were quite abundant in the CFL acid extract, their accumulation was less than 5 μg g^−1^ DM throughout the cell cycle (Table 1; Figure 6). This suggests a concentration-independent accumulation of REEs by *G. sulphuraria*.

**Table 1:** Concentration of individual REEs in a CFL acid extract.

REE	μg g^−1^ ± SD
Y	7,019 ± 280.76
La	10,387 ± 259.68
Ce	6,176 ± 222.34
Pr	0.3 ± 0.009
Nd	0.5 ± 0.0195
Sm	0.1 ± 0.004
Eu	594 ± 17.82
Gd	234 ± 5.85
Tb	2,929 ± 244.11
Dy	1.6 ± 0.08
Tm	0.2 ± 0.007
Yb	2.6 ± 0.065
Lu	39 ± 3.86

A one-way ANOVA was performed to compare the effect of time (different phases of the cell cycle-2 h, 10 h, 24 h) on the concentration of individual REEs in the biomass of *G. sulphuraria*. The analysis revealed a statistically significant difference in the concentration of individual REEs between at least two groups, for statistical values see Supplementary Table S2A. Tukey’s HSD test for multiple comparisons found that the mean value of the concentration of individual REEs was significantly different between the groups marked by asterisks in the Supplementary Table S2B. Overall, the concentration of all elements differed significantly between 2 h and 10 h and also between 2 h and 24 h, with the exception of two elements La and Ce, where the difference in their concentration was significant between all groups (2 h, 10 h, 24 h).

### The effect of plant hormones on REEs accumulation

**Figure 7:**
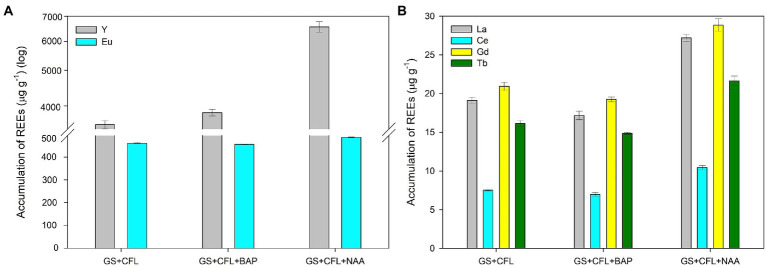
Effect of plant hormones NAA and BAP on the accumulation of individual REEs in *Galdieria sulphuraria* under CFL treatment. **(A)** Y and Eu accumulation following NAA and BAP treatments, and **(B)** La, Ce, Gd and Tb accumulation under NAA and BAP treatments. Expressed as concentrations in μg g^−1^. The error bars represent standard deviations (±SD). For details of statistical analysis (one-way ANOVA, Tukey’s HSD) see Supplementary Table S3.

Two synthetic plant hormones, i.e., BAP and NAA, were tested to determine their effect on the accumulation of REEs from the CFL acid extract. Before conducting this experiment, we studied the effect of these two hormones on cell shape, size, and growth of *G. sulphuraria*. Results showed that these hormones did not affect these parameters (Supplementary Figures S3, S4). The results showed that both hormones dramatically affected REE accumulation, but NAA had the more significant effect. The asynchronous culture of *G. sulphuraria*, after 24 h of growth, accumulated 3,596 and 319 μg g^−1^ DM of Y and Eu respectively, which increased to 6,556 (1.82-fold) and 451 (1.4-fold) μg g^−1^ DM, respectively, in the presence of NAA (Figure 7A). BAP also increased the accumulation of Y and Eu to 3,870 and 298 μg g^−1^ DM, respectively, (Figure 7A). Figure 7B shows the accumulation of La, Ce, Gd and Tb from the same experiment. NAA increased the accumulation of La from 19 to 27 μg g^−1^ DM, Ce from 7 to 10.5 μg g^−1^ DM, Gd from 20 to 29 μg g^−1^ DM and Tb from 16 to 21.5 μg g^−1^ DM (Figure 7B). However, BAP did not increase the accumulation of La, Ce, Gd, and Tb like Y and Eu (compare BAP in the Figures 7A,B). Compared to BAP, NAA had a more pronounced effect on the accumulation of REEs.

A one-way ANOVA was performed to compare the effect of treatment (CFL extract alone, CFL + BAP hormone, CFL + NAA hormone) on the concentration of individual REEs in the biomass of *G. sulphuraria*. The analysis revealed that there was a statistically significant difference in the concentration of individual REEs between at least two groups, for statistical values see Supplementary Table S3A. Tukey’s HSD test for multiple comparisons found that the mean value of the concentration of individual REEs was significantly different between the groups marked by asterisks in the Supplementary Table S3B. Altogether, valid for all elements, there was no significant difference in concentration of individual REEs in the biomass treated by CFL alone and CFL+ BAP. On the other hand, the concentration of REEs in the biomass treated by CFL + NAA was significantly different in comparison with the other two groups (CFL alone and CFL + BAP).

## Discussion

The extremophilic red alga *G. sulphuraria* was selected for the present study due to its ability to grow in a range of diverse habitats including those having thermophilic, acidophilic, halophilic and toxic metal conditions (Oesterhelt et al., 2007; Weber et al., 2007). The growth of cyanobacteria and algae in the presence of REEs has been demonstrated previously. Dubey and Dubey reported on the growth of three cyanobacteria, *Phormidium*, *Oscillatoria* and *Lyngbya*, in presence of red mud (Dubey and Dubey, 2011). Similarly, the growth of 6 living macroalgae species, i.e., *Ulva lactuca, Ulva intestinalis, Fucus spiralis, Fucus vesiculosus, Osmundea pinnatifida* and *Gracilaria* sp. in laboratory-prepared seawater solution containing REEs (Y, La, Ce, praseodymium (Pr), neodymium (Nd), Eu, Gd, Tb, dysprosium (Dy)) has been studied (Pinto et al., 2021). However, until now, only a few studies have been conducted to evaluate the growth and accumulation of REEs present in waste luminophores. Interestingly, this is the first study that examines the effect of a CFL acid extract on growth and the bioaccumulation of REEs present in electronic waste of CFL lights. Prior to this study, Čížková et al., used the unicellular red alga *Galdieria phlegrea* to examine the growth and bioaccumulation of REEs from luminophore powder (of two different sources such as energy saving light bulbs - CFL, and fluorescent lamps - FL) (Čížková et al., 2021). The findings of the present study confirmed that the growth of *G. sulphuraria* was optimal under the acidic conditions (Supplementary Figures S1, S2). Thus low growth (dry matter) observed in the presence of the CFL acid extract compared to the control culture (Figure 1) could have been due to a negative effect of REEs.

The biomass loss recorded in the dark phase of both control and treated cultures (Figure 1, dark phase) was due to the phenomenon of night biomass loss or respiration loss. This phenomenon is an essential property of photosynthetic algae, and acts as a tax on day biomass gains; approximately 30% of the algal biomass produced during the day can be lost at night (Guterman et al., 1989; Hu et al., 1998; Edmundson and Huesemann, 2015). Edmundson and Huesemann, studied night biomass loss in three potential commercial biomass-producing algal strains, *Chlorella sorokiniana*, *Nannochloropsis salina* and *Picochlorum* sp. They reported that specific night biomass loss rates were highly variable, and varied between −0.006 and −0.59 μ_dark_ day^−1^ (Edmundson and Huesemann, 2015). The result also showed that night biomass loss was species-specific and influenced by environmental conditions such as culture temperature and light intensity prior to and during the dark phase. The night biomass loss was positively corelated with increasing cultivation temperature (Torzillo et al., 1991; Edmundson and Huesemann, 2015). Given the high optimal cultivation temperature (40°C) of *G. sulphuraria*, loss of biomass during the dark phase was not surprising.

All three photosynthetic pigments increased with progression of the cell cycle in both control and CFL–treated cultures (Figure 2). However, the CFL–treated culture showed reduced levels of Chl *a* as compared to the control, probably due to adverse effects of REEs present in the CFL acid extract (Figure 2A). A negative effect of abiotic stresses on photosynthetic pigments of algae and cyanobacteria has also been reported (Sun et al., 2014; Fu et al., 2020). In the present experiment, the phycocyanin was least affected pigment by CFL acid stress (Figure 2C). The reason could be a high stability of phycocyanin of extremophilic *G. sulphuraria* which was significantly more stable compared to phycocyanin of common cyanobacteria *Spirulina platensis* (Wan et al., 2021). All the members of cyanidiophyceae were considered as best organisms for the production of stable phycobiliproteins (Carfagna et al., 2018; Ferraro et al., 2020). Regarding carotenoids, their function also comprises a defense mechanism to mitigate the damaging effects of stress in photosynthetic organisms (Shi et al., 2020; Potijun et al., 2021). Microalgae respond to an increased exposure to dissolved metals by accumulating carotenoids as antioxidant compounds (Gauthier et al., 2020). In the present study, carotenoid content increased during the initial 4 h of the cell cycle (Figure 2B), probably to mitigate the stress caused by prime accumulation of REEs (Figure 6; 2 h) in the CFL–treated culture. Nonetheless, due to increased accumulation of certain REEs (e.g., Y, Eu) with the progression of the cell cycle (Figure 6; 10 h, 24 h) and due to their toxicity, carotenoids were not able to cope with this stress and levels began to decrease (Figure 2B). It is known that under acute metal stress the antioxidant capacity of microalgae can be depleted (Pinto et al., 2003). Similarly to this study, a selenium (Se) concentration of up to 75 mg L^−1^ increased both the chlorophyll and carotenoid contents in *Chlorella vulgaris*; a higher concentration (>75 mg L^−1^) of Se caused a significant decline in the overall content of carotene and chlorophyll *a* (Sun et al., 2014). This result showed that if the level of stress was higher, then no defense mechanism of the cell could protect it. Similarly, Cheng et al., showed that an increasing concentration and exposure time to cadmium (Cd) can cause a decline in Chl *a*, Chl *b* and carotenoids in *C. vulgaris* (Cheng et al., 2016). Like other metal stresses, photosynthetic pigments of *Trachydiscus minutus* and *Parachlorella kessleri* were evaluated after exposure to several single REEs and monazite (Goecke et al., 2017). Results showed that pigment content was variable according to the element and algal species used.

The F_v_/F_m_ ratio signifies the maximum potential quantum yield of photosystem II, once all the reaction centers are exposed. It is used as an indicator of stress on PSII. Under stress conditions, the photosynthetic efficiency of PSII is reduced as the cell activates all photo-protective mechanisms. The determined starting values of F_v_/F_m_ ratio (0.58; 0.55) are typical for red algae (Oesterhelt et al., 2007; Iovinella et al., 2020), and are not comparable with green algae or plants due to a fluorescence interference of phycocyanin present in red algae. Our results showed that both the control and CFL–treated cultures expressed a fall in the F_v_/F_m_ ratio at the initial hour of the experiment, which could be due to sudden light exposure after a long (8 h) dark phase (Figure 3). Comparable F_v_/F_m_ values and pattern were determined during the cell cycle of *G. sulphuraria* treated with red mud extract (Náhlík et al., 2022). Fu et al. also observed a similar pattern in *Galdieria partita*, where the F_v_/F_m_ value started to decrease rapidly within 1 h of high light exposure (Fu et al., 2020). In our study, after the 1^st^ h of incubation, the control culture began to adapt to the new condition and eventually recovered significantly during the dark phase of the cell cycle. Nevertheless, CFL–treated cells were unable to recover significantly during the light phase of the experiment due to simultaneous light and CFL stress (Figure 3). Likewise, the maximum photochemical quantum yield (F_v_/F_m_) of the PSII reaction center decreased under La exposure in *D. quadricauda* (Ashraf et al., 2021). However, in *G. sulphuraria,* high light conditions stimulated cell growth and dry matter in both control and CFL–treated cultures. Recently, Kselíková et al., studied the effect of two different concentrations of deuterated water and three different light intensities on the F_v_/F_m_ ratio in *C. reinhardtii* and *D. quadricauda*. They observed reduced F_v_/F_m_ ratios with increasing light intensity and the concentration of deuterated water (Kselíková et al., 2022). The studies by Kselíková et al., and Ashraf et al., support results observed in the present study showing that increasing stress can reduce the photosynthetic efficiency of PSII (Ashraf et al., 2021; Kselíková et al., 2022).

Glycogen synthesis exactly followed the pattern of dry matter accumulation throughout the cell cycle in both control and CFL–treated cultures. Around 20–30% of the glycogen content produced in the light phase was consumed during the dark phase (Figure 4). Náhlík et al. observed a similar pattern of glycogen synthesis in synchronized cultures of *G. sulphuraria* (Náhlík et al., 2021). So far, the accumulation of glycogen during the cell cycle in *Galdieria* has not been studied. However, the consumption of glycogen during cell division was comparable to consumption of starch during the cell cycle in green algae (Vítová et al., 2015).

The algal cell cycle is associated with the nature of algal species and growth conditions. In the present study, the control culture of *G. sulphuraria* attained its 1^st^ division at 12 h and 2^nd^ at 16 h of the cell cycle (Figure 5A). Under the same growth conditions, *G. sulphuraria* attained the first cell division into two cells at 12 h and the second division into four cells at the 16th h of the cell cycle (Náhlík et al., 2021). Although, CFL–treated cells showed a 1–2 h delay in cell division which could be a toxicity adaptation time, eventually they completed their second division within the time course of the cell cycle (Figure 5B). At the end of the cell cycle (24 h of experiment) all cells had divided into four daughter cells in both control and CFL–treated cultures, but not released from the mother cell wall (Figures 5A,B). The 2 h shift was also confirmed by the calculation of the mass doubling time being about 10 h in the control culture and 12 h in the CFL treated culture. Although the cells finished the division to four in the CFL treated culture, the biomass gain was lower implying the daughter cell size had to be smaller (compare Figures 1, 5, 24 h). In contrast to *Cyanidioschyzon*, in *Galdieria* and *Cyanidium*, the divided cells (autospores) were surrounded by the mother cell wall before hatching (Jong et al., 2021). There was also a correlation between the number of following cell divisions and commitment cell size in cyanidialean red algae, including *G. sulphuraria*. The delayed cell division of the CFL–treated culture could be due to slower growth for cells to reach commitment size, as compared to the control culture (Jong et al., 2021). However, CFL–treated cells also finished the 2^nd^ division during the dark phase of the 24 h cell cycle. Similarly, *Pseudokirchneriella subcapitata*, a freshwater alga was exposed to different metals, i.e., cadmium (Cd), chromium (Cr) and copper (Cu), and their growth, cell volumes, and cell divisions were investigated over a period of 72 h (Machado and Soares, 2014). Results showed that the highest metal concentrations of Cr(VI) and Cu(II) arrested cell growth before the first nuclear division whereas Cd(II) arrested the cell after the second nuclear division but before the release of autospores from the mother cell wall (Machado and Soares, 2014). This variable impact of metals on the cell cycle of algae suggests that different metals trigger different toxicity mechanisms.

In the present study, REE accumulation was concentration-independent and highly selective with the progression of the cell cycle (Figure 6; Table 1). In *G. sulphuraria*, the accumulation of single REEs was not concentration dependent, e.g., the concentration of La and Ce was very high in the CFL acid extract, however the accumulation of Y and Eu was greater than the accumulation of La and Ce (Figures 6, 7). The increased or decreased metal concentration in cells at different phases of the cell cycle could be due to the selective behavior of *G. sulphuraria* towards individual REEs. If the cells were not able to tolerate toxicity of several REEs such as Ce and La, they excluded these metals from cells in a later phase of the cell cycle. This phenomenon could be a base for a future selective “extraction” of lanthanides from medium by continuous sampling during the cultivation. Similar findings were observed in *Galdieria phlegrea*, where the ratio of accumulated REEs differed significantly from their ratio in the growth medium (Čížková et al., 2021). Several studies show that the accumulation of light lanthanides, i.e., La, Ce, Pr, Nd, promethium (Pm), samarium (Sm) were preferred by organisms over heavy lanthanides (González et al., 2015; Yang and Wilkinson, 2018). Our results clearly showed that *G. sulphuraria* has tremendous capacity for lanthanide bio-mining/bio-removal, but further investigation is required to understand the detailed mechanisms behind the bioaccumulation/biosorption of REEs from e-waste (CFL lights) containing environments.

Interestingly, almost no research to date has been conducted on the effect of hormones on the bioaccumulation of REEs by microalgae. This is the first study that shows the accumulation of Y and Eu can be doubled in the presence of the plant hormones NAA and BAP (Figures 7A,B). However, the mechanism behind increased REE accumulation following NAA exposure remains elusive. A possible explanation could be that the application of exogenous cytokinins and auxins mitigated the toxicity of REEs and promoted cell growth, development, and regulated their adsorption similar to HM adsorption in green algae *C. vulgaris* and *Acutodesmus obliquus* (Piotrowska-Niczyporuk et al., 2012, 2018).

## Conclusion

Being acidophilic in nature, the red alga *G. sulphuraria* can grow in the presence of a CFL acid extract containing 10% HNO_3,_ although growth was slightly slower than the control culture. Photosynthetic pigments such as chlorophyll *a*, carotenoids and phycocyanin were also decreased under CFL treatment, although carotenoid synthesis minimized the deleterious effects of CFL up to the initial 4^th^ h of the cell cycle. During the light phase of the cell cycle, photosynthetic performance, expressed as the ratio F_v_/F_m_, was negatively affected by CFL treatment, but later in the dark phase, recovered significantly. In the initial hours of the cell cycle, cell division was delayed by about 2 h with CFL treatment, although later cells were able to complete their 2nd division within 24 h of the cell cycle. The produced daughter cells were of smaller size. Accumulation of REEs by *G. sulphuraria* was concentration-independent and selective. The REEs most accumulated by *G. sulphuraria* were Y and Eu, followed by La, Ce, Gd, and Tb, respectively. The plant hormone NAA stimulated the accumulation of REEs and almost doubled Y accumulation. BAP also had a pronounced effect on Y and Eu accumulation, although it did not increase the accumulation of other REEs. Waste luminophores like CFL bulbs could be a great secondary source of REEs, and using the red alga *G. sulphuraria* may be promising in REE bio-removal/accumulation technology. Recovery of REEs from algal biomass could lead to an economic and eco-friendly solution for the removal of hazardous waste from CFL lights and for the generation of REEs for future use.

## Data availability statement

The original contributions presented in the study are included in the article/Supplementary material, further inquiries can be directed to the corresponding author.

## Author contributions

MV, DS, and DM: conceptualization and funding acquisition. AS, MČ, and VN: methodology, investigation, and writing—original draft preparation. DS and DM: validation. MR: formal analysis. AS and MČ: resources. MV and DM: data curation, writing—review and editing, and project administration. MV and MR: visualization. MV: supervision. All authors contributed to the article and approved the submitted version.

## Funding

This research was funded by the European fund for regional development, the program Interreg V-A Austria – Czech Republic, the Project ATCZ172 REEgain, the COST Action 19116 – PLANTMETALS and by the institutional support RVO 61388971 and RVO 67985939 of the Czech Academy of Sciences.

## Conflict of interest

The authors declare that the research was conducted in the absence of any commercial or financial relationships that could be construed as a potential conflict of interest.

## Publisher’s note

All claims expressed in this article are solely those of the authors and do not necessarily represent those of their affiliated organizations, or those of the publisher, the editors and the reviewers. Any product that may be evaluated in this article, or claim that may be made by its manufacturer, is not guaranteed or endorsed by the publisher.

## Footnotes

^1^ http://www.acuf.net/index.php?lang=en

^2^ https://www.real-statistics.com/free-download/real-statistics-resource-pack/
