# Supplementary material for: Bio-removal of rare earth elements from hazardous industrial waste of CFL bulbs by the extremophile red alga Galdieria sulphuraria
Source: Front Microbiol. 2023 Feb 13;14:1130848. doi: 10.3389/fmicb.2023.1130848 (PMC9969134; doi:10.3389/fmicb.2023.1130848)
Supplement: Supplementary file 1 [file Table_1.pdf]

Supplementary Table S1 Multiple linear regression - β coefficients and p-values for Figures 2 - 4.

	Chlorophyll *a*		Carotenoids		Phycocyanin		F_v/F_m ratio		Glycogen	
coefficient name	coeff.	p-value	coeff.	p-value	coeff.	p-value	coeff.	p-value	coeff.	p-value
β1= Intercept	0.72	0.000193	29.15	0.076888	71.48	4.33E-07	0.51	1.11E-09	0.88	0.021825
β2= Time	0.2	2.09E-06	23.48	3.35E-06	12.41	2.1E-07	-0.03	0.003914	0.67	1.18E-10
β3= Treatment	-0.24	0.129863	21.94	0.252945	-14.21	0.067905	-0.17	0.007807	-1.03	0.023216
β4= Time^2	-0.003	0.001522	-0.53	0.000245	-0.32	6.36E-06	0.001	0.000435	-0.02	2.33E-08
β5= Time*Treat	-0.045	0.001695	-4.94	0.003252	0.05	0.912711	0.001	0.816817	-0.09	0.003562

Statistically significant p-values < 0.05 are highlighted in red.
