# Supplementary material for: Bio-removal of rare earth elements from hazardous industrial waste of CFL bulbs by the extremophile red alga Galdieria sulphuraria
Source: Front Microbiol. 2023 Feb 13;14:1130848. doi: 10.3389/fmicb.2023.1130848 (PMC9969134; doi:10.3389/fmicb.2023.1130848)
Supplement: Supplementary file 2 [file Table_2.pdf]

Supplementary Table S2 One-way ANOVA (panel A) and Tukey's HSD test (panel B) for Figure 6.

A)

REE	F value (ANOVA)	p-value
Y	F (2, 6) = 538923.9	1.72E-16
La	F (2, 6) = 1186.3	1.60E-08
Ce	F (2, 6) = 1775.9	4.79E-09
Pr	F (2, 6) = 1862.9	4.15E-09
Nd	F (2, 6) = 3312.7	7.40E-10
Sm	F (2, 6) = 3327.2	7.31E-10
Eu	F (2, 6) = 950.3	3.11E-08
Gd	F (2, 6) = 2896.3	1.10E-09
Tb	F (2, 6) = 945.8	3.16E-08

B)

group 1	group 2	**Yttrium**				**Lanthanum**			
		mean diff.	95% C.I.		p-value	mean diff.	95% C.I.		p-value
time1	time2	*761.15	697.71	824.59	1.09E-07	*19.69	17.69	21.68	2.58E-07
time1	time3	*703.95	640.50	767.39	1.59E-07	*24.64	22.64	26.63	9.33E-08
time2	time3	57.21	-6.24	120.65	0.073	*4.95	2.96	6.94	6.50E-04
		**Cerium**				**Praseodymium**			
		mean diff.	95% C.I.		p-value	mean diff.	95% C.I.		p-value
time1	time2	*97.70	90.39	105.01	5.97E-08	*12.93	12.18	13.69	7.68E-09
time1	time3	*99.05	91.74	106.36	5.47E-08	*12.94	12.19	13.69	7.66E-09
time2	time3	1.35	-5.96	8.67	0.840	0.0029	-0.75	0.75	0.999
		**Neodymium**				**Samarium**			
		mean diff.	95% C.I.		p-value	mean diff.	95% C.I.		p-value
time1	time2	*46.89	44.17	49.61	7.64E-09	*12.02	11.49	12.54	1.45E-10
time1	time3	*46.86	44.14	49.58	7.69E-09	*12.01	11.49	12.54	1.45E-10
time2	time3	0.03	-2.69	2.75	0.999	0.002	-0.52	0.52	0.999
		**Europium**				**Gadolinium**			
		mean diff.	95% C.I.		p-value	mean diff.	95% C.I.		p-value
time1	time2	*142.03	129.05	155.02	1.69E-07	*10.81	10.05	11.57	3.92E-08
time1	time3	*114.60	101.62	127.59	4.17E-07	*10.94	10.18	11.70	3.59E-08
time2	time3	*27.43	14.45	40.41	1.56E-03	0.14	-0.62	0.89	0.852
		**Terbium**							
		mean diff.	95% C.I.		p-value				
time1	time2	*1.27	1.16	1.37	1.16E-07				
time1	time3	*1.30	1.19	1.40	1.02E-07				
time2	time3	0.03	-0.08	0.14	0.653				

Tukey's HSD test of REE concentration expressed as 95% confidence intervals (C.I.) of the mean difference (mean diff.) between groups indicated as time1 (2 h), time2 (10 h), and time3 (24 h). The effect of time on the concentration of every single REE in the biomass of *G. sulphuraria* is shown separately. A difference in group means equal to zero indicates that the group means are equal, so only a confidence interval that does not contain zero is statistically significant (alpha = 0.05, indicated by an asterisk). Statistically significant p-values < 0.05 are highlighted in red.
