# Supplementary material for: Bio-removal of rare earth elements from hazardous industrial waste of CFL bulbs by the extremophile red alga Galdieria sulphuraria
Source: Front Microbiol. 2023 Feb 13;14:1130848. doi: 10.3389/fmicb.2023.1130848 (PMC9969134; doi:10.3389/fmicb.2023.1130848)
Supplement: Supplementary file 3 [file Table_3.pdf]

Supplementary Table S3 One-way ANOVA (panel A) and Tukey's HSD test (panel B) for Figure 7.

A)

REE	F value (ANOVA)	p-value
Y	F (2, 6) = 268.13	1.35E-06
Eu	F (2, 6) = 80.63	4.61E-05
La	F (2, 6) = 253.87	1.59E-06
Ce	F (2, 6) = 195.39	3.46E-06
Gd	F (2, 6) = 151.09	7.38E-06
Tb	F (2, 6) = 140.83	9.07E-06

B)

group 1	group 2	Yttrium				Europium			
		mean diff.	95% C.I.		p-value	mean diff.	95% C.I.		p-value
CFL	CFL+BAP	2745.87	-1587.79	7079.53	0.207	207.20	-192.00	606.41	0.319
CFL	CFL+NAA	*29599.65	25265.99	33933.31	1.78E-06	*1316.08	916.88	1715.28	1.34E-04
CFL+BAP	CFL+NAA	*26853.78	22520.13	31187.45	3.28E-06	*1523.28	1124.08	1922.49	5.79E-05
		**Lanthanum**				**Cerium**			
		mean diff.	95% C.I.		p-value	mean diff.	95% C.I.		p-value
CFL	CFL+BAP	1.96	-1.45	5.37	0.182	0.55	-0.03	1.13	0.060
CFL	CFL+NAA	*8.04	4.64	11.45	4.54E-03	*2.92	2.34	3.49	1.14E-05
CFL+BAP	CFL+NAA	*10.01	6.60	13.41	2.40E-03	*3.46	2.89	4.04	4.03E-06
		**Gadolinium**				**Terbium**			
		mean diff.	95% C.I.		p-value	mean diff.	95% C.I.		p-value
CFL	CFL+BAP	1.68	-0.13	3.49	0.065	1.26	-0.06	2.58	0.059
CFL	CFL+NAA	*7.91	6.10	9.72	2.62E-05	*5.51	4.20	6.83	3.38E-05
CFL+BAP	CFL+NAA	*9.59	7.78	11.40	8.44E-06	*6.77	5.46	8.09	1.02E-05

Tukey's HSD test of REE concentration expressed as 95% confidence intervals (C.I.) of the mean difference (mean diff.) between groups indicated as treatment1 (CFL), treatment2 (CFL+BAP), and treatment3 (CFL+NAA). The effect of treatment on the concentration of every single REE in the biomass of *G. sulphuraria* is shown separately. A difference in group means equal to zero indicates that the group means are equal, so only a confidence interval that does not contain zero is statistically significant (alpha = 0.05, indicated by an asterisk). Statistically significant p-values < 0.05 are highlighted in red.
