# Supplementary material for: Bio-removal of rare earth elements from hazardous industrial waste of CFL bulbs by the extremophile red alga Galdieria sulphuraria
Source: Front Microbiol. 2023 Feb 13;14:1130848. doi: 10.3389/fmicb.2023.1130848 (PMC9969134; doi:10.3389/fmicb.2023.1130848)
Supplement: Supplementary file 4 [file Image_1.pdf]

Supplementary Figure S1

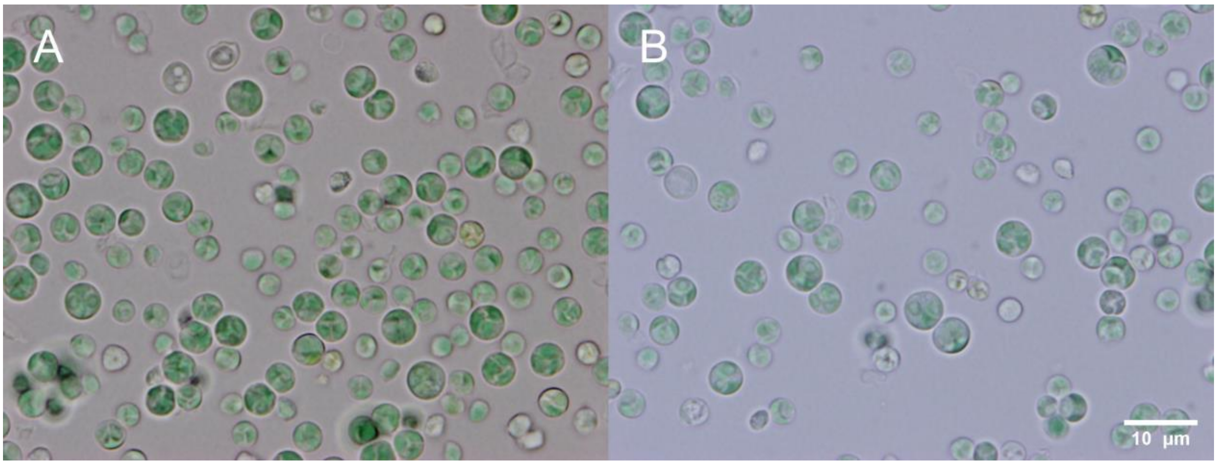

Photomicrographs showing the effect of 10% HNO_3 on *Galdieria sulphuraria* after 24 h of growth. Control (A), treated with 10% HNO_3 (B). Results showed no negative effect of 10% HNO_3. The bar is 10 µm.
