# Supplementary material for: Bio-removal of rare earth elements from hazardous industrial waste of CFL bulbs by the extremophile red alga Galdieria sulphuraria
Source: Front Microbiol. 2023 Feb 13;14:1130848. doi: 10.3389/fmicb.2023.1130848 (PMC9969134; doi:10.3389/fmicb.2023.1130848)
Supplement: Supplementary file 5 [file Image_2.pdf]

Supplementary Figure S2

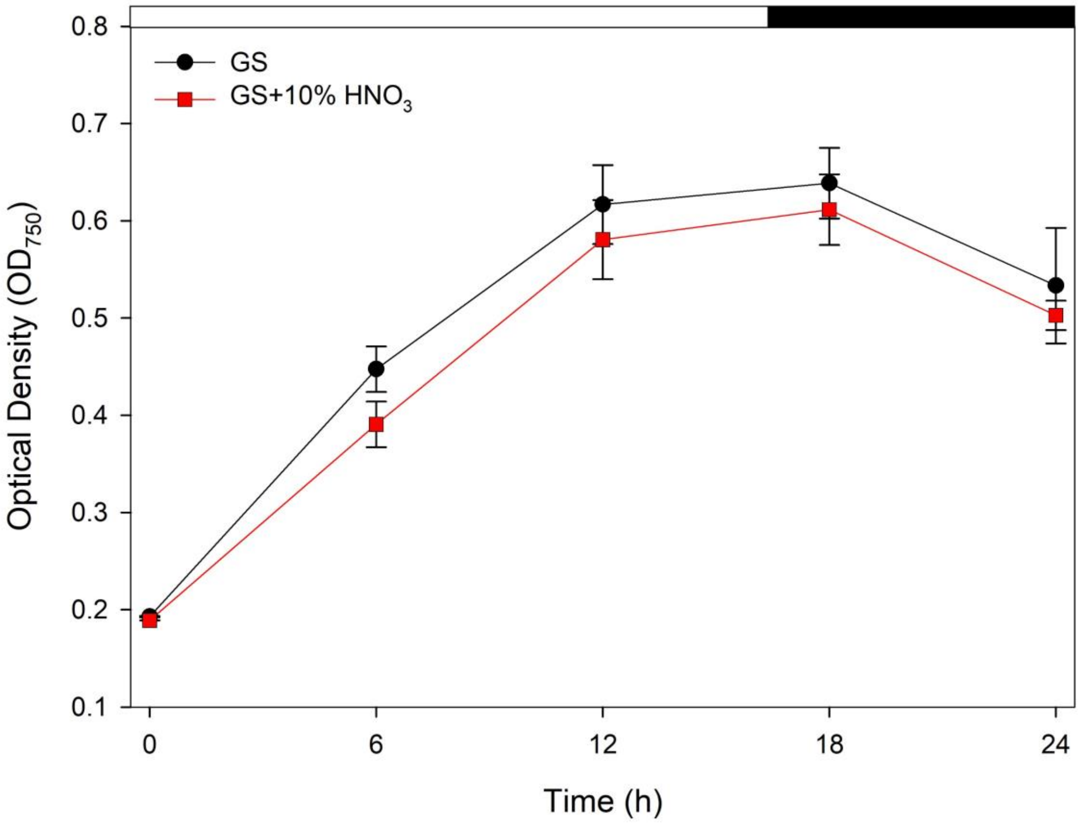

Growth curves of *Galdieria sulphuraria* measured as Optical Density (OD_{750}) showing the effect of 10% HNO_3. Control (black circles), treated with 10% HNO_3 (red squares).
