# Supplementary material for: Bio-removal of rare earth elements from hazardous industrial waste of CFL bulbs by the extremophile red alga Galdieria sulphuraria
Source: Front Microbiol. 2023 Feb 13;14:1130848. doi: 10.3389/fmicb.2023.1130848 (PMC9969134; doi:10.3389/fmicb.2023.1130848)
Supplement: Supplementary file 6 [file Image_3.pdf]

**Supplementary Figure S3**

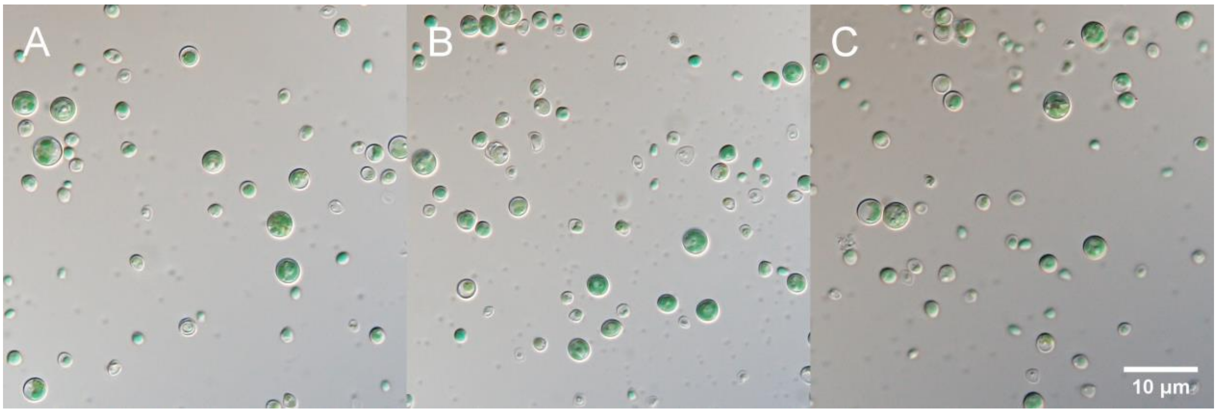


Photomicrographs showing the effect of hormones on *Galdieria sulphuraria*. Control (A), treated with NAA (B), treated with BAP (C). Results showed no negative effect of the hormones. The bar is 10 µm.
