# Supplementary material for: Bio-removal of rare earth elements from hazardous industrial waste of CFL bulbs by the extremophile red alga Galdieria sulphuraria
Source: Front Microbiol. 2023 Feb 13;14:1130848. doi: 10.3389/fmicb.2023.1130848 (PMC9969134; doi:10.3389/fmicb.2023.1130848)
Supplement: Supplementary file 7 [file Image_4.pdf]

Supplementary Figure S4

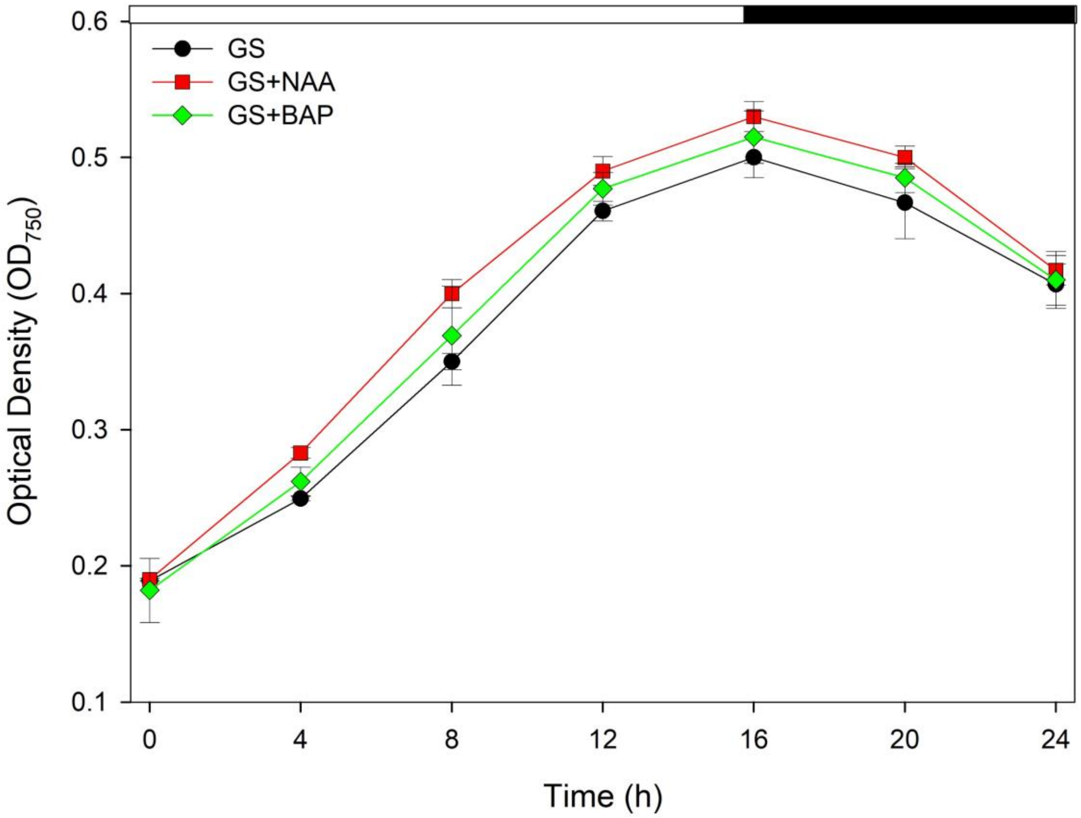

Growth curves of *Galdieria sulphuraria* measured as Optical Density (OD_{750}) showing the effect of two hormones i.e. NAA and BAP. Control (black circles), treated with NAA (red squares), treated with BAP (green diamonds).
